# 伴Ph阳性附加染色体异常慢性髓性白血病的生物学特征及疗效分析

**DOI:** 10.3760/cma.j.issn.0253-2727.2021.08.008

**Published:** 2021-08

**Authors:** 晓燕 董, 玉龙 李, 成业 邬, 保军 商, 琳 张, 薇 程, 尊民 朱

**Affiliations:** 河南省人民医院血液病研究所，河南省血液病理重点实验室，河南省干细胞分化与调控重点实验室，郑州大学人民医院，河南大学人民医院，郑州 450003 Institute of Hematology, Henan Provincial People's Hospital; Henan Key Laboratory of Hematopathology; Henan Key Laboratory of Stem Cell Differentiation and Modification, People's Hospital of Zhengzhou University; People's Hospital of Henan University, Zhengzhou 450003, China

**Keywords:** 附加染色体, 白血病，髓性，慢性，BCR-ABL阳性, 预后, Additional chromosomal abnormalities, Leukemia, myelogenous, chronic, BCRABL positive, Prognosis

## Abstract

**目的:**

探讨Ph阳性附加染色体异常（ACA/Ph^+^）对初诊慢性期（CP）和治疗中进展为加速期和急变期慢性髓性白血病（CML-AP/BP）患者生物学特征、疗效和预后的影响。

**方法:**

回顾性分析2013年1月至2020年6月河南省人民医院收治的410例Ph^+^ CML[初诊CML-CP 348例，治疗中进展为AP/BP（进展期CML）62例]患者的临床资料，根据ELN2020标准将其分为高危、非高危和无ACA/Ph^+^三组，并比较分析高危/非高危ACA/Ph^+^对其生物学特征、疗效和预后的影响。

**结果:**

①348例初诊CML-CP患者，合并ACA/Ph^+^者20例（5.75%），其中高危ACA/Ph^+^组3例，非高危ACA/Ph^+^组17例；无ACA/Ph^+^组328例。伴ACA/Ph^+^和无ACA/Ph^+^组患者的基本临床特征差异无统计学意义（*P*值均>0.05）；非高危ACA/Ph^+^组和无ACA/Ph^+^组间完全血液学缓解（CHR）率、完全细胞遗传学反应（CCyR）率、主要分子学反应（MMR）率和5年总生存（OS）率差异均无统计学意义（*P*值均>0.05）；非高危ACA/Ph^+^组5年无进展生存（PFS）率显著低于无ACA/Ph^+^组（42.0%对74.5%，*χ*^2^＝4.766，*P*＝0.029）。②62例进展期CML患者，合并ACA/Ph^+^者41例（66.13%），其中高危ACA/Ph^+^组28例，非高危ACA/Ph^+^组13例；无ACA/Ph^+^组21例。高危ACA/Ph^+^组患者中位PLT水平（42.5×10^9^/L）低于非高危（141×10^9^/L）和无ACA/Ph^+^组（109×10^9^/L）（*χ*^2^＝4.968，*P*＝0.083）；三组间ABL激酶区点突变发生率差异无统计学意义（*P*＝0.652）。高危ACA/Ph^+^组CCyR率显著低于无ACA/Ph^+^组（5.3%对46.7%，*χ*^2^＝5.851，*P*＝0.016）。高危ACA/Ph^+^组5年OS率为46.2%，非高危ACA/Ph^+^组为64.3%，无ACA/Ph^+^组为77.8%，其中高危ACA/Ph^+^组患者5年OS率明显低于无ACA/Ph^+^组（*χ*^2^＝3.878，*P*＝0.049）。亚组分析显示高危Ⅰ组（+8，+Ph或含+8/+Ph的复杂ACA）CML患者的5年OS率为54.5%，与无ACA/Ph^+^组相比差异无统计学意义（*χ*^2^＝1.514，*P*＝0.219）；高危Ⅱ组[含−7/7q−或i（17q）或含2个及以上高危ACA的复杂核型]为28.6%，显著低于无ACA/Ph^+^组（*χ*^2^＝8.035，*P*＝0.005）。

**结论:**

因ACA类型和疾病分期不同，伴ACA/Ph^+^ CML患者的治疗反应和预后存在差异，治疗过程中高危ACA的出现意味着更差的治疗反应和预后，严格、规范的细胞遗传学监测对此类患者的早期发现和精准诊疗具有重要意义。

## Abstract (en)

**Objective:**

To investigate the effects of additional chromosomal abnormalities (ACA) in Philadelphia chromosome-positive (Ph^+^) cells on biological characteristics, therapy efficacy, and prognosis of patients with primary chronic myeloid leukemia (CML)-chronic phase (CP) and those who developed CML-accelerated phase/blast phase (AP/BP) during therapy.

**Methods:**

The clinical data of 410 patients with Ph^+^ CML, including 348 patients with primary CML-CP and 62 patients who progressed to CML-AP/BP during treatment, who were admitted to Henan People's Hospital from January 2013 to June 2020 were retrospectively analyzed to categorize into high-risk, non-high-risk, and non-ACA groups according to the ELN2020 criteria. The effects of high- and non-high-risk ACA on biological characteristics, therapy efficacy, and prognosis were compared.

**Results:**

① Among the 348 patients with primary CML-CP, 20 patients (5.75%) had ACA, including 3 and 17 patients with high-risk and non-high-risk ACA, respectively, whereas the remaining 328 patients did not have ACA. There were no significant differences in baseline clinical characteristics between those with and without ACA (*P*>0.05 for all). The rates of complete hematological response, complete cytogenetic response, major molecular remission, and 5-year overall survival (OS) were not significantly different between the non-high-risk ACA and non-ACA groups (*P*>0.05 for all) ; however, the 5-year progression-free survival of the non-high-risk ACA group (42.0%) was significantly lower than that of the non-ACA group (74.5%) (*χ*^2^=4.766, *P*=0.029).② Of the 62 patients who progressed to CML-AP/BP during treatment, 41 patients (66.13%) had ACA, including 28 and 13 patients with high-risk and non-high-risk ACA, respectively, whereas the remaining 21 patients did not have ACA. Platelet counts of the high-risk ACA group (42.5×10^9^/L) were lower than those of the non-high-risk (141×10^9^/L) and non-ACA groups (109×10^9^/L) (*χ*^2^=4.968, *P*=0.083). There was no significant difference in the incidence of point mutations in ABL kinase among the three groups (*P*=0.652). The complete cytogenetic response of the high-risk ACA group (5.3%) was significantly lower than that of the non-ACA group (46.7%) (*χ*^2^=5.851, *P*=0.016). The 5-year OS of the high-risk ACA group was lower than that of the non-ACA group (46.2% *vs* 77.8%, *χ*^2^=3.878, *P*=0.049). Subgroup analysis revealed that the 5-year OS rate of the high-risk group Ⅱ, which included −7/7q−, i (17q), and complex karyotype comprising ≥2 high-risk ACA, was significantly lower than that of the non-ACA group (28.6% *vs* 77.8%, *χ*^2^=8.035, *P*=0.005) whereas the 5-year OS rate was not significantly different between high-risk group Ⅰ, which included +8,+Ph, and complex ACA with +8/+Ph, and the non-ACA group (54.5% *vs* 77.8%, *χ*^2^ =1.514, *P*=0.219).

**Conclusion:**

Due to different disease stages and ACA/Ph^+^ types, treatment response and prognosis vary among patients with CML harboring ACA/Ph^+^. The emergence of high-risk ACA during therapy suggests worse therapy efficacy and prognosis. Strict and standardized cytogenetic monitoring is critical for early detection, precise diagnosis, and treatment of these patients.

慢性髓性白血病（CML）是一种以Ph染色体和（或）BCR-ABL融合基因阳性为特征的骨髓增殖性肿瘤。尽管酪氨酸激酶抑制剂（TKI）靶向治疗时代，CML患者的预后有了很大改善，但仍有部分患者疗效欠佳。研究发现，5%~10%的初诊CML慢性期（CP）、30%的加速期（AP）和50%~80%的急变期（BP）患者可在Ph阳性细胞中出现附加染色体异常（ACA/Ph^+^）[1]。通常认为ACA/Ph^+^是患者遗传不稳定、疾病进展的标志[2]。随着对ACA/Ph^+^研究的进一步深入，越来越多的学者认为ACA/Ph^+^出现的时期及其类型等与CML患者预后密切相关[3]–[5]。欧洲白血病网（ELN）2020标准将+8、+Ph、i（17q）、+19、−7/7q−、11q23、3q26.2和复杂核型定义为高危ACA/Ph^+^[1]。本研究中我们回顾性分析了本中心410例Ph^+^CML患者的临床资料，着重探讨高危和非高危ACA/Ph^+^对不同疾病状态下CML患者预后和生存的影响。

## 病例与方法

1．病例资料：2013年1月至2020年6月于我院就诊的初诊CML-CP患者348例，其中伴ACA/Ph^+^者20例；进展期CML（治疗中进展为AP/BP）患者62例，其中伴ACA/Ph^+^者41例。CML诊断、治疗及随访参照文献[6]–[7]。311例采用TKI治疗的CML患者的临床资料可供疗效和预后分析。

2．染色体核型分析：骨髓染色体核型分析采用短期培养法，常规制备染色体标本并进行R显带处理。核型异常按《人类细胞遗传学国际命名体制（ISCN，2005）》描述。根据ELN 2020标准将+8、+Ph、i（17q）、+19、−7/7q−、11q23、3q26.2和复杂核型划分为高危ACA组[1]，其余ACA/Ph^+^为非高危ACA组。无ACA/Ph^+^为无ACA组。结合文献[4]–[5]，本研究将高危ACA组进一步划分为高危Ⅰ组和高危Ⅱ组：+8、+Ph或含+8/+Ph的复杂ACA为高危Ⅰ组，含−7/7q−、i（17q）或2个及以上高危ACA的复杂核型为高危Ⅱ组。

3．分子生物学检测：BCR-ABL融合基因检测采用实时荧光定量PCR法，结果以国际标准化（IS）值的形式表示。ABL激酶区点突变检测采用PCR荧光探针法，检测位点包含Y253H、E255K/V、V299L、T315A、T315I、F317V/I/C/L、F359V/C/I。检测试剂盒分别购自上海源奇生物医药科技有限公司和苏州云泰生物医药科技有限公司，检测详细步骤参照说明书。

4．TKI治疗反应、预后评估及随访：完全血液学缓解（CHR）、完全细胞遗传学反应（CCyR）和主要分子学反应（MMR）的定义参照文献[1],[6]。总生存（OS）时间定义为自疾病诊断至死亡或随访终止的时间，无进展生存（PFS）时间定义为自疾病诊断至进展、死亡或随访终止的时间。进展期CML患者自确诊疾病进展为AP/BP开始收集基线资料。采用查阅病历或电话的方式随访。随访截止时间为2020年9月1日，中位随访时间39（1~97）个月。

5．统计学处理：应用SPSS23.0软件进行统计学分析，计量资料两组间比较采用*t*检验、Mann-Whitney *U*检验，三组间比较采用Kruskal-Wallis *H*检验；计数资料组间比较采用*χ*^2^检验或Fisher精确检验。生存分析采用Kaplan-Meier法，组间比较采用Log-rank检验。*P*<0.05为差异有统计学意义。

## 结果

一、初诊CML-CP患者合并ACA情况、临床特征及疗效、预后分析

1．初诊CML-CP患者合并ACA情况及其临床特征：348例初诊Ph^+^ CML-CP患者，合并ACA 20例（5.75%），其中高危ACA组3例：2例单独+8，1例复杂核型；非高危ACA组17例，其中包含染色体数目异常5例，平衡易位2例和其他结构异常10例；无ACA组328例。将高危与非高危ACA组合并为ACA组，其与无ACA组患者的基本临床特征见表1。两组患者间性别、年龄、WBC、HGB、PLT、ELTS（EUTOS long-term survival，EUTOS长期生存）评分差异均无统计学意义（*P*值均>0.05）。

**表1:** 伴与不伴Ph阳性附加染色体异常（ACA）初诊慢性髓性白血病慢性期患者的基本临床特征比较

临床特征	ACA组（20例）	无ACA组（328例）	统计量	*P*值
性别（例，男/女）	14/6	209/119	0.323	0.570
年龄[岁，*M*（范围）]	49（13~73）	44（1~90）	−1.008	0.314
WBC[×10^9^/L，*M*（范围）]	116.1（18.9~320.0）	171.3（10.1~681.8）	−1.008	0.314
HGB[g/L，*M*（范围）]	101（63~171）	102（46~164）	0.594	0.553
PLT[×10^9^/L，*M*（范围）]	450（168~1866）	409（68~3766）	1.227	0.220
ELTS评分[例（%）]				0.812
低危	15（75.0）	215（65.5）		
中危	4（20.0）	85（25.9）		
高危	1（5.0）	28（8.5）		

2．初诊CML-CP合并ACA患者的ABL激酶区突变情况：266例初诊CML-CP患者有随访资料，ACA组17例（高危ACA组1例，非高危ACA组16例），无ACA组249例。ACA组仅4例非高危ACA患者行ABL激酶区点突变检测，均为阴性；74例无ACA组患者检测ABL激酶区点突变，阳性14例，包括E255K/V 11例、Y253H 2例、F317V/I/C/L联合V299L 1例。

3．疗效与预后评估：初诊CML-CP有随访资料的高危ACA组患者仅1例，该患者规律服用伊马替尼，治疗25个月达MMR，因仅为个例，故暂无法评价高危ACA对初诊CML-CP患者疗效和预后的影响。本研究仅对非高危ACA组和无ACA两组CML-CP患者的疗效和预后进行分析：两组CHR率、治疗12个月时CCyR率、总的累积CCyR率和MMR率分别为93.8%对96.4%、81.2%对81.5%、87.5%对91.2%、75.0%对73.5%，差异均无统计学意义（*P*值均>0.05）。进一步比较两组患者的长期生存，非高危ACA组和无ACA组CML-CP患者5年OS率分别为92.9%和97.2%，组间差异无统计学意义（*χ*^2^＝1.003，*P*＝0.303）（图1）；两组患者5年PFS率分别为42.0%和74.5%，非高危ACA组显著低于无ACA组（*χ*^2^＝4.766，*P*＝0.029）（图2）。

**图1:**
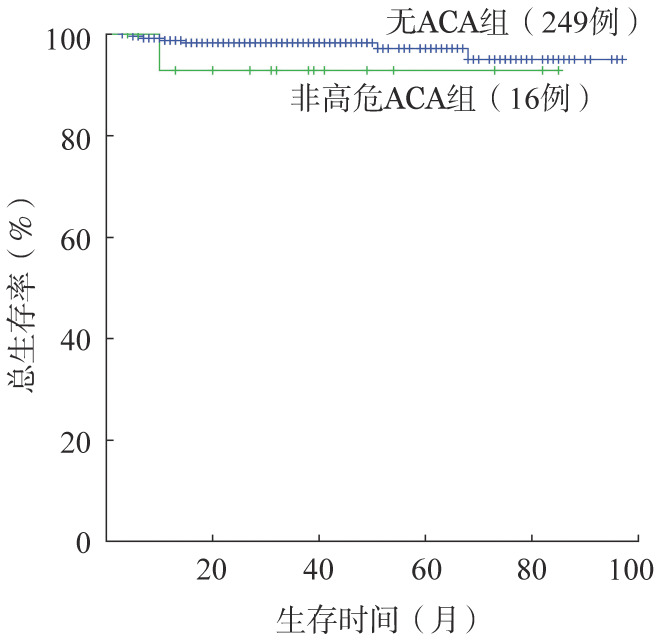
初诊慢性期慢性髓性白血病非高危与无Ph阳性附加染色体异常（ACA）组患者总生存的比较

**图2:**
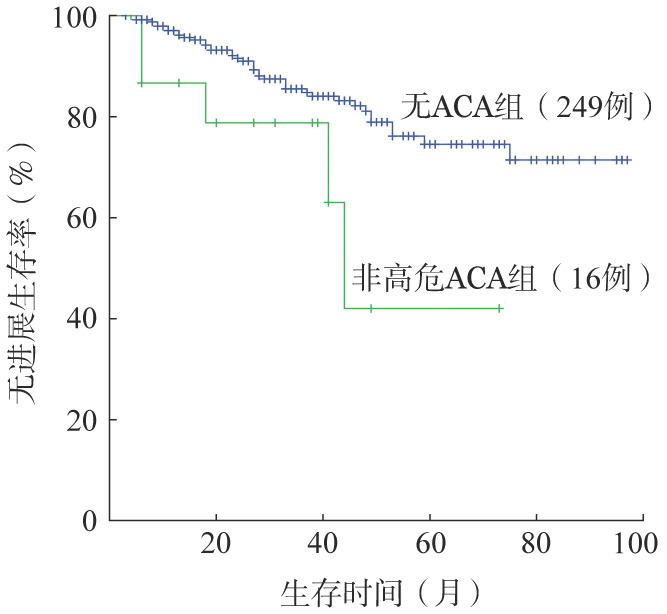
初诊慢性期慢性髓性白血病非高危与无Ph阳性附加染色体异常（ACA）组患者无进展生存的比较

二、进展期CML患者合并ACA情况、临床特征及疗效、预后分析

1．进展期CML患者合并ACA情况及其临床特征：进展期CML患者62例，其中合并ACA者41例（66.13%）：高危ACA组28例，包含复杂核型24例，+Ph 11例，+8 8例，+19 3例，−7/7q− 3例，i（17q） 2例；非高危ACA组13例，包含染色体数目异常1例，平衡易位1例和其他结构异常11例；无ACA组21例。三组患者的基本临床特征见表2。比较三组患者的性别、年龄、WBC、HGB及诊断分期，差异均无统计学意义（*P*值均>0.05）。高危ACA组患者中位PLT水平（42.5×10^9^/L）明显低于非高危（141×10^9^/L）和无ACA组（109×10^9^/L）（*χ*^2^＝4.968，*P*＝0.083）。

**表2:** 伴高危、非高危和不伴Ph阳性附加染色体异常（ACA）进展期CML患者的基本临床特征比较

临床特征	高危ACA组（28例）	非高危ACA组（13例）	无ACA组（21例）	统计量	*P*值
性别（例，男/女）	16/12	7/6	14/7	0.685	0.710
年龄[岁，*M*（范围）]	54（12~67）	46（23~61）	42（16~70）	0.416	0.812
WBC[×10^9^/L，*M*（范围）]	28.5（0.7~359.6）	30.2（2.6~174.0）	30.9（1.3~409.5）	0.293	0.864
HGB[g/L，*M*（范围）]	74.5（49~124）	88（45~116）	80.96（30~126）	1.538	0.464
PLT[×10^9^/L，*M*（范围）]	42.5（2~770）	141（8~688）	109（5~2362）	4.968	0.083
诊断分期[例（%）]				2.172	0.337
CML-AP	15（53.6）	5（38.5）	7（33.3）		
CML-BP	13（46.4）	8（61.5）	14（66.7）		

注：CML-AP：慢性髓性白血病加速期；CML-BP：慢性髓性白血病急变期

2．进展期CML合并ACA患者的ABL激酶区突变情况：45例进展期CML患者临床资料可供分析。高危ACA组19例，12例行ABL激酶区点突变检测，其中阳性者5例：E255K/V 3例、T315I 2例；非高危组11例，8例接受ABL激酶区点突变检测者4例阳性：Y253H及E255K/V联合突变1例、F359V/I/C/L、F317I和T315I各1例；无ACA组15例，11例行ABL激酶区点突变检测，3例阳性，分别为E255K/V 2例，Y253H 1例。三组间ABL激酶区点突变发生率差异无统计学意义（*P*＝0.652）。

3．疗效和预后评估：进展期CML高危ACA组患者19例，仅1例（5.3%）患者由一代TKI更换为二代TKI联合化疗方案治疗后在12个月内达到CCyR；非高危ACA组和无ACA组患者治疗12个月CCyR率则分别为9.1%和46.7%；总的累积CCyR率三组分别为5.3%、36.4%和46.7%，高危ACA组CCyR率与无ACA组相比差异具有统计学意义（*χ*^2^＝5.851，*P*＝0.016），非高危ACA组和无ACA组差异无统计学意义（*χ*^2^＝0.015，*P*＝0.902）。进一步比较三组患者的长期生存，5年OS率：高危ACA组为46.2%，非高危ACA组为64.3%，无ACA组为77.8%（*χ*^2^＝4.355，*P*＝0.113），其中高危ACA组患者5年OS率明显低于无ACA组（*χ*^2^＝3.878，*P*＝0.049）（图3）；三组患者的5年PFS率分别为28.8%、35.0%和50.4%，差异无统计学意义（*χ*^2^＝0.916，*P*＝0.633）。

**图3:**
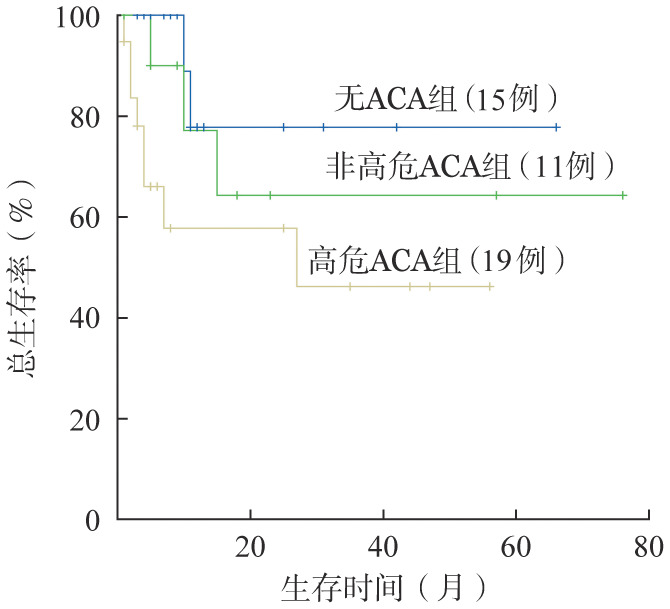
进展期慢性髓性白血病高危、非高危和无Ph阳性附加染色体异常（ACA）组患者总生存的比较

将高危ACA组分为高危Ⅰ组（+8，+Ph或含+8/+Ph的复杂ACA）和高危Ⅱ组[含−7/7q−，i（17q）或含2个及以上高危ACA的复杂核型]，其中高危Ⅰ组患者的5年OS率为54.5%，与无ACA组相比差异无统计学意义（*χ*^2^＝1.514，*P*＝0.219）；高危Ⅱ组5年OS率为28.6%，显著低于无ACA组，差异具有统计学意义（*χ*^2^＝8.035，*P*＝0.005）（图4）。

**图4:**
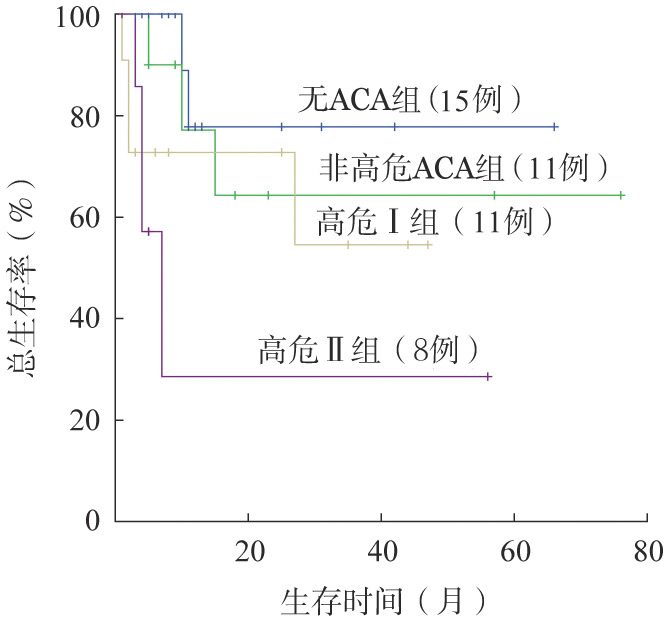
进展期慢性髓性白血病高危Ⅰ、高危Ⅱ、非高危和无Ph阳性附加染色体异常（ACA）组患者总生存的比较

## 讨论

通常情况下，ACA/Ph^+^发生在CML初诊、治疗失败或疾病进展时，是疾病由CP进入AP/BP的重要驱动因素。在TKI治疗时代，多数学者认为合并ACA/Ph^+^与CML的病情进展密切相关，尤其是高危类型ACA[8]–[9]。ELN2020标准将+8、+Ph、i（17q）、+19、−7/7q−、11q23、3q26.2和复杂核型定义为高危ACA/Ph^+^，认为高危ACA/Ph^+^意味着更差的治疗反应和更高的疾病进展风险[1]。本研究着重分析高危、非高危ACA/Ph^+^对不同疾病状态下CML患者治疗反应和预后的影响。

本研究中348例初诊CML-CP患者中合并ACA者20例（5.75%）；62例进展期CML患者中41例（66.13%）合并ACA，ACA的发生频率与既往报道一致，另外，ACA/Ph^+^最常见的类型为+Ph、+8，其次为+19、i（17）和−7/7q−，也与既往文献报道相一致[5],[10]–[11]。此外，初诊CML-CP组合并复杂核型ACA者显著少于进展期CML患者，可能因后者先前治疗药物的暴露，诱发了基因的不稳定，从而导致更多ACA的发生[2]。

临床特征方面，因初诊CML-CP合并高危ACA病例过少，将高危和非高危ACA组合并为ACA组，两组间基本临床特征差异均无统计学意义；进展期CML中高危ACA组患者具有相对较低的PLT水平（42.5×10^9^/L），但差异无统计学意义（*P*＝0.083），其他临床特征间差异亦无统计学意义。

关于ACA与CML患者ABL激酶区突变的关联仍有争议：Savasoglu等[12]的研究显示ACA与CML患者ABL激酶区突变无明显相关性；Willis等[13]对CML发生ABL激酶区突变进行多因素分析，结果显示ACA/Ph^+^与之密切相关。我们的结果显示无论初诊CML-CP或进展期CML，ACA与无ACA组ABL激酶区突变率差异均无统计学意义，且高危/非高危ACA也与ABL激酶区突变无显著相关性。突变类型上，似乎初诊CML-CP患者无ACA组E255K/V更常见，而T315I突变类型更常发生在进展期CML患者中，但由于本研究纳入行ABL激酶区点突变检测的CML患者较少，各组是否在突变位点分布上存在差异尚需更多的临床数据予以证实。

ACA的存在与CML患者（主要是CP）治疗反应、生存期的关联亦存争议：Alhuraiji等[14]的研究显示初诊CML伴ACA/Ph^+^患者的累积CCyR率、累积MMR率、OS及EFS等与经典易位Ph^+^患者相比差异均无统计学意义；也有学者认为合并高危类型ACA CML患者的CCyR率和MMR率较经典易位CML患者更低，达到CCyR和MMR所需的时间也更长，OS更差[4]–[5],[8]；Wang等[5]报道预后不良ACA[单独i（17q）、−7/7q−、3q26.2]对进展期CML患者的生存期无显著影响。

我们在研究中对初诊CML-CP患者非高危ACA与无ACA组的CHR率、CCyR率和MMR率进行比较，两组间未见明显差异；5年OS率差异也无统计学意义；非高危ACA组5年PFS率显著低于无ACA组，与文献报道一致[15]–[17]，提示初诊CML-CP患者合并ACA/Ph^+^可能并不影响OS，但ACA组患者在治疗过程中可能更易发生不良事件，随访中应适当增加病情监测频率，必要时及时更改治疗策略。由于本研究中初诊CML-CP患者中高危ACA组病例过少，故无法评估高危ACA对此类患者治疗反应和预后的影响，有待更多数据的积累或多中心大样本的研究。

对治疗中进展为AP/BP的患者进行分析，结果显示高危ACA组患者CCyR率显著低于无ACA组，高危ACA组5年OS率明显低于其他两组，与既往文献报道一致[4],[16],[18]。结合文献[4],[5]，i（17q）、−7/7q−和3q26.2无论作为单独的ACA/Ph^+^或复杂ACA的一部分，都预示着疾病的快速进展和更差的预后；而单独+8和+Ph较其他高危ACA具有相对良好的预后。本研究将高危ACA组进一步划分为高危Ⅰ组和高危Ⅱ组，亚组分析显示高危Ⅱ组具有更差的OS。该结果提示不同类型的高危ACA预后意义仍有差异，对高危ACA进一步亚组分析有助于更高危CML患者的识别和更精准地指导治疗。

综上所述，在CML初诊及治疗过程中常发生ACA/Ph^+^，因ACA类型及出现时机不同，其对CML患者疗效和预后的影响亦不相同，应根据相关指南加强对CML患者进行细胞遗传学监测，并根据疾病状态对ACA进行准确分类，以便及时调整治疗策略，取得更好的临床获益。
